# Role of a bacterial glycolipid in Sec-independent membrane protein insertion

**DOI:** 10.1038/s41598-022-16304-1

**Published:** 2022-07-18

**Authors:** Kaoru Nomura, Shoko Mori, Kohki Fujikawa, Tsukiho Osawa, Shugo Tsuda, Kumiko Yoshizawa-Kumagaye, Shun Masuda, Hideki Nishio, Taku Yoshiya, Takao Yoda, Masafumi Shionyu, Tsuyoshi Shirai, Ken-ichi Nishiyama, Keiko Shimamoto

**Affiliations:** 1 Bioorganic Research Institute, Suntory Foundation for Life Sciences, 8-1-1 Seikadai, Seika-cho, Soraku-gun, Kyoto, 619-0284 Japan; 2 Department of Chemistry, Graduate School of Science, Osaka University, 1-1 Machikaneyama, Toyonaka, Osaka 560-0043 Japan; 3 Peptide Institute, Inc., 7-2-9 Saito-Asagi, Ibaraki, Osaka 567-0085 Japan; 4 Department of Frontier Bioscience, Nagahama Institute of Bio-Science and Technology, 1266 Tamura-cho, Nagahama, Shiga 526-0829 Japan; 5 Department of Biological Chemistry and Food Sciences, Faculty of Agriculture, Iwate University, 3-18-8 Ueda, Morioka, Iwate 020-8550 Japan

**Keywords:** Biophysics, Biotechnology

## Abstract

Non-proteinaceous components in membranes regulate membrane protein insertion cooperatively with proteinaceous translocons. An endogenous glycolipid in the *Escherichia coli* membrane called membrane protein integrase (MPIase) is one such component. Here, we focused on the Sec translocon-independent pathway and examined the mechanisms of MPIase-facilitated protein insertion using physicochemical techniques. We determined the membrane insertion efficiency of a small hydrophobic protein using solid-state nuclear magnetic resonance, which showed good agreement with that determined by the insertion assay using an in vitro translation system. The observed insertion efficiency was strongly correlated with membrane physicochemical properties measured using fluorescence techniques. Diacylglycerol, a trace component of *E. coli* membrane, reduced the acyl chain mobility in the core region and inhibited the insertion, whereas MPIase restored them. We observed the electrostatic intermolecular interactions between MPIase and the side chain of basic amino acids in the protein, suggesting that the negatively charged pyrophosphate of MPIase attracts the positively charged residues of a protein near the membrane surface, which triggers the insertion. Thus, this study demonstrated the ingenious approach of MPIase to support membrane insertion of proteins by using its unique molecular structure in various ways.

## Introduction

**Figure 1:**
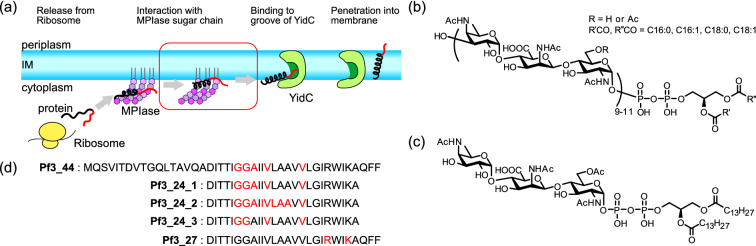
(**a**) Schematic of the pathways involving post-translational Sec-independent protein insertion into the inner membrane (IM) of the *E. coli*. Small membrane protein is released from a ribosome and binds to the sugar chains of membrane protein integrase (MPIase). MPIase prevents the protein from aggregating and alters the secondary structure of the protein. The hydrophobic region of protein proceeds into the membrane. Subsequently, the hydrophilic region binds to the hydrophilic groove (dark green) of an insertase, YidC (green) and is further translocated across the periplasmic membrane leaflet. In this study, we focused on the process outlined in red in the figure. (**b**) Molecular structure of MPIase. Approximately one-third of the 6-position on GlcNAc residues is *O*-acetylated. The number of repeating trisaccharide units ranges from 7 to 14, but most are from 9 to 11. (**c**) Mini-MPIase-3, which is a synthetic analog. (**d**) Amino acid sequence and variation of the selective ^15^N label of substrate protein used in this study: full-length Pf3 coat protein Pf3_44, and its substructures, Pf3_24_1, Pf3_24_2, Pf3_24_3, and Pf3_27. ^15^N-labeled amino acids are colored in red.

Membrane protein insertion is a crucial aspect of cell membrane biology, with a fundamental process common to prokaryotic and eukaryotic cells. A large number of membrane proteins are co-translationally inserted into membranes with the assistance of Sec translocons (Sec-dependent pathway)^1–3^. In contrast, some small membrane proteins containing one or two transmembrane domain(s) followed by a short C-terminus are inserted post-translationally without the help of translocons (Sec-independent pathway) (Fig. 1a)^4,5^. While constructing an in vitro integration system, Nishiyama et al*.* found that artificial liposomes that are composed of commercially available *E. coli* phospholipids (EPLs), which contain only phosphatidylethanolamine (PE), phosphatidylglycerol (PG), and cardiolipin (CL), could not reproduce the Sec-dependency of proteins observed in *E. coli* inner membrane vesicles (INVs), leading to the discovery of two insertion-regulating factors in the INVs^6,7^. The first is an insertion-inhibiting factor, diacylglycerol (DAG), a minor non-phospholipid component of the *E. coli* membrane. A physiological concentration of DAG blocks the unregulated spontaneous membrane insertion of proteins into EPL liposomes^7^. In addition, an insertion-promoting factor was discovered, where the blocked insertion in DAG-containing EPL liposomes was restored by adding an extract from INVs, suggesting the presence of the membrane protein integrase (MPIase)^6^. DAG and MPIase are required to regulate both Sec-dependent and independent pathways^6^. Despite its name, MPIase was determined to be a novel glycolipid through structural analysis^8^. It has a sugar chain that comprises approximately 10 repeating trisaccharide units and a DAG anchor connected via a pyrophosphate linker, as shown in Fig. 1b. The purified MPIase drove Sec-independent insertion in DAG-containing liposomes in a dose-dependent manner^9^. We synthesized a minimal structural unit of MPIase, mini-MPIase-3 (Fig. 1c), consisting of only one trisaccharide unit^10^. Mini-MPIase-3 showed significant activity, although it is less efficient than MPIase, suggesting that it includes an essential structure for membrane insertion^10^.

In general, Sec-independent insertion is assisted by the insertase, YidC^11–13^. The hydrophilic region of a substrate protein is transiently captured in the hydrophilic groove of YidC within the membrane^14^. Local thinning of lipid bilayers due to short transmembrane domains of YidC and/or membrane potential enable the hydrophilic regions of the protein to cross the bilayer^11,15–17^. Our recent study revealed that the protein released from the ribosome is captured by sugar chains of MPIase and attracted to the membrane surface through the pyrophosphate group^18^. In the presence of MPIase, some hydrophobic proteins such as the 3L-Pf3 coat, a mutant version of bacteriophage Pf3 coat protein, are inserted into the membrane without YidC, while YidC accelerates the insertion in combination with MPIase^19,20^. Therefore, we speculated that the hydrophobic region of the protein would be inserted into the membrane and the hydrophilic region would be delivered into YidC after attraction by MPIase.

In this study, we examined how MPIase supports the insertion process using solid-state nuclear magnetic resonance (NMR) and fluorescence techniques. Our recent study demonstrated that inverted cone-shaped MPIase (Fig. S3) and cone-shaped DAG altered the membrane physicochemical properties, including the lipid acyl chain ordering, membrane packing, membrane surface flexibility, membrane lateral diffusion, and flip-flop motion of DAG^21^. In this study, we evaluated how the MPIase- and DAG-induced changes in physicochemical properties regulate the insertion of a transmembrane domain of the membrane protein by comparing the correlation between the membrane physicochemical properties and the insertion efficiency to those in various types of membranes. Then, the topologies of the transmembrane domain of the protein inside the lipid bilayer in the presence and absence of MPIase were compared. Membrane proteins are inserted into the membrane at certain insertion angles^22,23^, but the angle sometimes changes depending on coexisting phospholipids^24^. The unique molecular shape of MPIase could change the protein conformation in the membrane, as MPIase has a large head group (Fig. 1b). Moreover, we investigated the intermolecular interaction between a pyrophosphate in mini-MPIase-3 and arginine (Arg) and lysine (Lys) sidechains in the protein embedded in EPL membranes. Most Sec-independent small membrane proteins contain positively charged residues at the sequence located in the cytoplasm, and these residues are important for targeting the negatively charged membrane surface, especially in *E. coli* membranes, through electrostatic interactions^25^. Therefore, the double negatively charged pyrophosphate might interact more strongly with the basic amino acids in proteins than the monophosphate of other phospholipids.

Consequently, our results provide a detailed mechanism of MPIase-facilitated membrane insertion in Sec-independent pathways. MPIase attracts the protein to the membrane surface and allows the protein to easily gain access to the membrane core by changing the membrane core environment. This is the first study to reveal the molecular interaction between MPIase and substrate proteins in the lipid membrane environment.

## Results

### Topology of the inserted substrate protein in oriented membranes

We examined the influence of MPIase on the tilt angle of a substrate protein with respect to the membrane. The Pf3 coat protein (Fig. 1d) is widely used as a model substrate protein in studies on the Sec-independent membrane protein insertion^4^. However, as outlined in the Introduction, YidC and/or membrane potential is required for the hydrophilic N-terminal region to penetrate the membrane completely^5,11^. In fact, Pf3_44 is hardly inserted into the 1,2-dimyristoyl-*sn*-glycero-3-phosphocholine (DMPC)/1,2-diheptanoyl-*sn*-glycero-3-phosphocholine (DHPC) bicelle ([DMPC]/[DHPC] = 3), as shown in Fig. S4a. Alternatively, in this study, we used only the transmembrane region of Pf3 coat protein, Pf3_24 (Fig. 1d), because it is likely to penetrate the membrane without the help of YidC or membrane potential due to its high hydrophobicity.

First, we tried to insert ^15^N-labeled Pf3_24, Pf3_24_1 (Fig. 1d) into the DMPC/DHPC bicelle system since it was reported that spontaneous insertion into PC liposomes was more efficient than that into EPL liposomes^26^ and the DMPC/DHPC bicelle system was more stable than the EPL/DHPC system^27^. Pf3_24_1 was inserted into the DMPC/DHPC bicelle system to provide signals in the 1D ^15^N spectrum (Fig. S4b), corresponding to spontaneous insertion in the absence of an inhibitory molecule (e.g., DAG).

**Figure 2:**
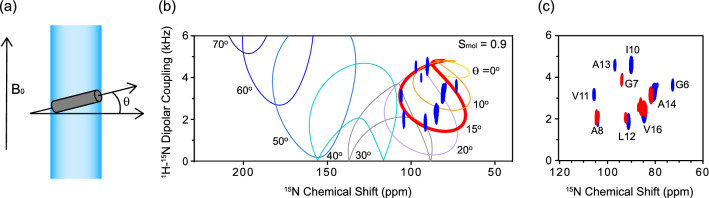
(**a**) Schematic of the magnetically aligned lipid bilayer (blue) and inserted Pf3_24_2 (gray). θ is the angle formed between the helix long axis of Pf3_24_2 and the membrane normal. (**b**) ^1^H-^15^N SAMPI4 spectra of Pf3_24_2 (Fig. 1d) in the DMPC/DHPC bicelles ([DMPC]/[DHPC] = 3) aligned with their normal perpendicular to the magnetic field. The simulated PISA wheels with varied tilt angles θ (0–70°) are overlaid onto the spectra. The bicelle order parameter S_mol_ is fixed at 0.9. (**c**) ^1^H-^15^N SAMPI4 spectra of Pf3_24_2 in the absence (blue) and presence (red) of 2 mol% of mini-MPIase-3.

Subsequently, we examined the structure and topology of Pf3_24 after membrane insertion using ^1^H-^15^N SAMPI4 spectra. In the SAMPI4 spectra, generally the ^15^N chemical shift is correlated with ^1^H-^15^N dipolar coupling and the *α*-helical part of the protein exhibits characteristic wheel-like resonance patterns, called the PISA wheel^28^, where the shape and size depend on the orientation angle of the helix in the magnetic field (Fig. 2a and b)^29,30^. Therefore, the tilt angle of the protein with respect to membranes can be obtained using aligned bicelles. In this experiment, we used Pf3_24_2, with ^15^N-labeled Pf3_24 at nine residues (Fig. 1d).

**Figure 3:**
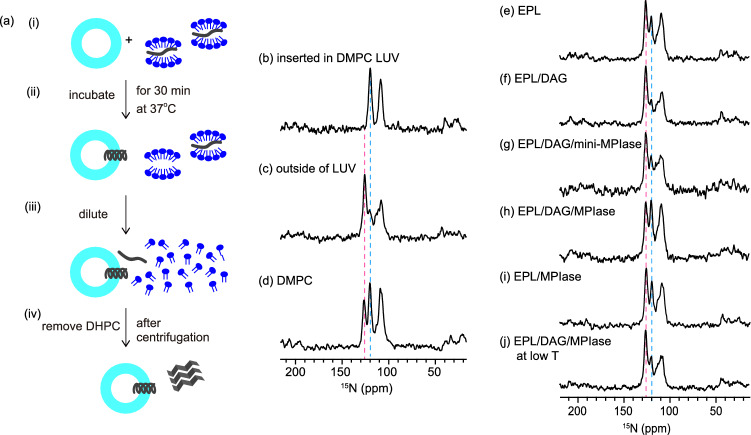
(**a**) Procedure for inserting Pf3_24_3 (Fig. 1d) into membranes. (i) Pf3_24_3 (dark gray) solubilized in a DHPC solution was mixed with LUVs composed of various types of lipids. (ii) The mixture was incubated for 30 min at 37 °C. (iii) Then, it was diluted with a buffer solution until the concentration of DHPC reached below the critical micelle concentration of DHPC (1.6 mM). (iv) The supernatant containing DHPC was removed after centrifugation and this step was repeated two times to remove DHPC. We confirmed that Pf3_24_3 outside the membrane was completely precipitated at this stage and not present in the supernatant (Fig. S5). The precipitates were subjected to ^15^N CPMAS NMR. (**b**–**j**) ^15^N CPMAS NMR spectra of Pf3_24_3. (**b**) Pf3_24_3 reconstituted into DMPC LUV. The membrane insertion procedure shown in (**a**) was omitted for this sample. (**c**) Pf3_24_3 in the aggregates. The sample was prepared as shown in (**a**) but without using LUVs. Therefore, all proteins were recovered as aggregates. (**d**–**j**) The sample was prepared as shown in (**a**). The LUVs were composed of DMPC (**d**), EPL (**e**), EPL/5 mol% DAG (**f**), EPL/5 mol% DAG/5 mol% mini-MPIase-3 (**g**), and EPL/5 mol% DAG/1 mol% MPIase (**h**), EPL/1 mol% MPIase (**i**). (**j**) The lipid composition was similar to (**h**), but all membrane insertion processes were performed below 4 °C. The pink and light blue dashed lines show the chemical shift at 125.6 and 119.6 ppm, respectively.

The SAMPI4 spectrum of Pf3_24_2 in the bicelle showed a characteristic PISA wheel pattern (Fig. 2b)^29,31^, where the resonances were then compared to the simulation patterns. The motion of bicelles (S_mol_) was considered because bicelles have a rapid and restricted “wobble” motion^32^. Spectrum fitting with S_mol_ = 0.9^32^ revealed that Pf3_24_2 was inserted into the membrane with a tilt angle of approximately 15° away from the lipid bilayer normal (Fig. 2b). Figure 2c shows the comparison of the ^1^H-^15^N SAMPI4 spectra in the presence and absence of 2 mol% of mini-MPIase-3 in the bicelles. The resonances for G6, I10, V11, and A13 weakened in the presence of mini-MPIase-3. However, other resonances appeared at approximately similar frequencies, indicating that mini-MPIase-3 had a limited effect on the tilt angle of Pf3_24_2. The preparation of other bicelle samples composed of various lipids was difficult because of their instability. Therefore, we changed the protocol and used large unilamellar vesicles (LUVs) for further experiments (Fig. 3a).

### Membrane insertion of Pf3_24 into the DMPC membrane

We determined the membrane insertion efficiency of Pf3_24 into LUVs using the 1D ^15^N cross polarization (CP) spectra under magic angle spinning (MAS). It is known that the chemical shifts of ^15^N NMR strongly correlate with the secondary structure^33,34^. Wang and Jardetzky^33^ reported that the averaged ^15^N chemical shift values of valine and glycine residues were 123.27 and 110.19 ppm for the *β*-strand, 119.66 and 109.94 ppm for the random coil, and 119.53 and 107.34 ppm for the *α*-helix, respectively. Therefore, we first estimated the secondary structures inside and outside the membrane using Pf3_24_3 (Fig. 1d), with ^15^N-labeled Pf3_24 (G6, G7, V11, and V16). Figure 3b shows the ^15^N CP MAS NMR spectrum of Pf3_24_3, which was reconstituted into DMPC LUV. The chemical shift values of valine and glycine were approximately 120 and 107 ppm, respectively, suggesting that Pf3_24_3 completely adopted the *α*-helical structure inside the LUVs, as expected. However, when Pf3_24_3 was added to the buffer without liposomes, valine signal appeared notably at 125 ppm in addition to that at 120 ppm (Fig. 3c). Most of the proteins were aggregated in the aqueous environment under this condition and the signal at 125 ppm indicates that Pf3_24_3 formed a *β*-strand-rich structure in the aggregates. The smaller signal at 120 ppm might originate from a random-coil conformation in addition to an *α*-helix conformation. The ratio of these conformations in the aggregates remained constant, as the relative peak intensities at 125 and 120 ppm were almost constant in the repeated experiments (Table 1). Glycine signals were observed between 114 and 107 ppm. Valine signals were used to predict the conformation of the protein in this experiment because they are fully separated and more decisive than glycine signals. Moreover, the spectrum is the sum of the peaks of the inserted proteins (Fig. 3b) and the aggregated proteins outside the membrane (Fig. 3c) if Pf3_24_3 added from outside (Fig. 3a) is partially inserted into the membrane. Relative peak intensities at 125 ppm (*I*^125^) and 120 ppm (*I*^120^) are expressed as follows:

1 $$I^{125}:I^{120} = xI^{125}_{{{\text{in}}}} + \left( {100 - x} \right)I^{125}_{{{\text{out}}}}:xI^{120}_{{{\text{in}}}} + \left( {100 - x} \right)I^{120}_{{{\text{out}}}}$$

where *x* is the membrane insertion efficiency (%) of Pf3_24_3, *I*^125^_in_ and *I*^120^_in_ are the relative peak intensities when Pf3_24_3 is completely inserted (Fig. 3b), and *I*^125^_out_ and *I*^120^_out_ are the relative peak intensities of the aggregates (Fig. 3c). Table 1 lists the following values used for the calculation of Eq. (1): *I*^125^_in_ = 0, *I*^125^_out_ = 77, *I*^120^_in_ = 100, and *I*^120^_out_ = 23.

**Table 1:** Relative signal intensities of valine residues at 125 ppm (*I*^125^) and 120 ppm (*I*^120^) shown in Fig. 3b and c. Mean values ± standard from three independent experiments.

	*I*^125^	*I*^120^
Inside the membrane	0 ± 0	100 ± 0
Outside the membrane	77 ± 2	23 ± 2

*I*^120^ shown in Fig. 3d is larger than that in Fig. 3c, when Pf3_24_3 was added to the DMPC LUV. Therefore, it was considered that Pf3_24_3 was partially inserted into the DMPC membrane, although it was added from outside the membrane. Using Eq. (1), it was determined that approximately 46 ± 1.7% of Pf3_24_3 was inserted into the DMPC membrane (Table 2). The small measurement error indicates that the insertion efficiency was almost constant.

**Table 2:** Relative signal intensities at 125 ppm (*I*^125^) and 120 ppm (*I*^120^) shown in Fig. 3d–i, and the insertion efficiency of Pf3_24, *x*, into the various membranes.

	*I*^125^	*I*^120^	Insertion efficiency *x* (%)^a^
DMPC	42	58	46 ± 1
EPL	59	41	24 ± 2
EPL/DAG (95/5)	76	24	2 ± 1
EPL/DAG/mini-MPIase-3 (90/5/5)	66	34	15 ± 3
EPL/DAG/MPIase (94/5/1)	50	50	36 ± 2
EPL/MPIase (99/1)	39	61	26 ± 2

^a^*x* values were calculated using Eq. (1). Errors were estimated from the noise in each spectrum in Fig. 3d–i using the method described in Supplementary Methods.

### Effect of DAG and MPIase on EPL membrane insertion of Pf3_24

We applied the protocol to obtain the insertion efficiency to the EPL LUVs (Fig. 3e). Since Pf3_24_3 formed the *α*-helical structure completely inside the EPL LUVs (Fig. S6), the same values as the DMPC LUVs (*I*^125^_in_ = 0 and *I*^120^_in_ = 100) can be used for the EPL LUVs. Valine signals were observed in the same chemical shifts described above at a ratio of 59:41, indicating that approximately 24% of Pf3_24_3 was inserted into the EPL membrane (Table 2). We evaluated the effect of DAG as an insertion inhibitory factor. The insertion efficiency decreased to almost zero in the presence of 5 mol% of DAG (Fig. 3f) in the EPL LUVs, demonstrating the potent inhibitory activity of DAG. In contrast, the addition of 5 mol% of mini-MPIase-3 or 1 mol% of MPIase into the DAG-containing EPL LUV (Fig. 3g and h) restored the insertion efficiency to 15% and 36%, respectively. Interestingly, the addition of 1 mol% of MPIase into DAG-free EPL LUV increased membrane insertion by only 2% (Fig. 3i and Table 2). These results highlighted the potency of MPIase to selectively counteract the effects of DAG. MPIase led to higher insertion than mini-MPIase-3 did, even though its concentration was five times lower. The sugar chain length of MPIase was approximately 10 times longer than mini-MPIase-3, as shown in Fig. 1b and c. Therefore, the longer sugar chain in MPIase enhanced the membrane insertion probability of Pf3_24_3. The results are in good agreement with the protein insertion assay using an in vitro translation system^8–10^. Thus, the applicability of this approach for determining membrane insertion efficiency was verified. *I*^120^ significantly decreased when the entire process of the EPL/DAG/MPIase LUV below 4 °C was performed, as shown in Fig. 3j. In this condition, the insertion should be blocked due to the high-lipid chain order at a low temperature.

### Correlation between membrane physicochemical property and membrane insertion efficiency of Pf3_24

Small hydrophobic proteins have been reported to be spontaneously inserted into vesicles composed of bulk membrane phospholipids without DAG^35,36^. Therefore, we further determined the insertion efficiency for several membranes composed of various types of bulk lipids (Fig. S7 and Table S1). We chose 1,2-dimyristoyl-*sn*-glycero-3-phospho-(1′-rac-glycerol) (DMPG) and 1-palmitoyl-2-oleoyl-*sn*-glycero-3-phospho-L-serine (POPS) to compare the effects of negative charge, and 1-palmitoyl-2-oleoyl-*sn*-glycero-3-phosphoethanolamine (POPE) to compare the effects of the headgroup size on DMPC. Additionally, we examined PE/PG-mixed membranes because EPL is mainly composed of these components. The insertion efficiency did not depend on the negative charge of the phospholipids but decreased with increasing PE content in PE/PG-mixed membranes. Addition of CL, which possesses double negative charges, to the PE/PG-mixed membrane increased the insertion efficiency slightly.

**Figure 4:**
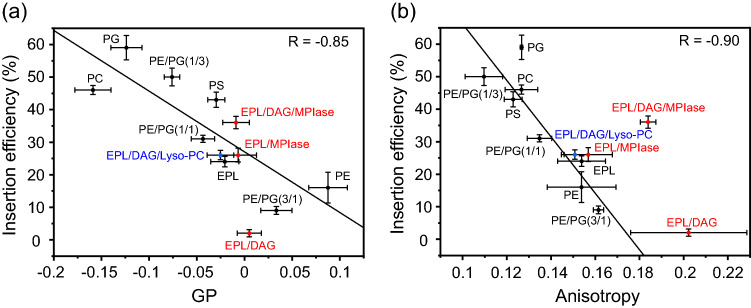
Correlation between membrane insertion efficiency values (Tables 2 and S1) and Laurdan GP values at 37 °C, $GP = \left( {I_{440} - I_{490} } \right)/\left( {I_{440} + I_{490} } \right)$, calculated from the emission spectra (Fig. S8) (**a**), and membrane insertion efficiency values and fluorescence anisotropy values of DPH at 37 °C (Fig. S9) (**b**) in the various types of membranes. LUVs that are composed of bulk membrane phospholipids are represented by black circles in each figure, whereas red circles represent the LUVs including DAG and/or MPIase. Blue circles represent the LUVs including Lyso-PC. Global linear fits were performed for the bulk phospholipids shown in black.

We also investigated the membrane surface packing and mobility of acyl chains in the membrane core region in various membranes to estimate the contribution of their physicochemical properties to membrane insertion. The membrane surface packing was analyzed by measuring the emission spectra of 6-lauroyl-2-dimethylamino naphthalene (Laurdan) (Fig. S8). Figure 4a (black circles) shows the plot of insertion efficiency against the generalized polarization (GP) values at 37 °C, which exhibited a significant correlation (*R* = -0.85). Regarding the PE/PG-mixed membranes, the membrane packing became considerably tighter as the PE concentration increased (Fig. S8b). The mobility of acyl chains was measured by the anisotropy of the fluorescent probe 1,6-diphenyl-1,3,5-hexatriene (DPH), which was incorporated near the bilayer core region (Fig. S9). The anisotropy values were also strongly correlated with membrane insertion (*R* = -0.90) (Fig. 4b, black circles). The black line shows the correlation between the insertion efficiency of bulk membrane lipids and the packing (Fig. 4a) or membrane anisotropy (Fig. 4b). The values for EPL membranes containing DAG (5 mol%) and/or MPIase (1 mol%) are plotted in red. It is apparent that DAG and MPIase influenced DPH anisotropy more than the GP value of Laurdan did. DAG did not affect the surface packing but strongly reduced acyl chain mobility in the membrane core region. In the absence of DAG, MPIase hardly affected either the insertion efficiency or membrane properties. However, in the presence of DAG, MPIase partially restored the mobility in the membrane core. Thus, MPIase counteracted the effects of DAG on the membrane properties.

The inverted cone shape of MPIase contributed to the altered membrane property. Therefore, we examined Lyso-PC that also has an inverted shape. The addition of 5 mol% of Lyso-PC to the DAG-containing EPL LUVs enhanced the insertion by 24% (Fig. S11a and Table S2). The values for Lyso-PC were plotted on Fig. 4 in blue. The anisotropy of EPL/DAG/5 mol% Lyso-PC LUVs was almost completely restored to that of EPL LUVs. As the spot is almost on the regression lines (Fig. 4), the Lyso-PC-enhanced insertion can be explained only by the alteration in membrane property caused by the inverted cone shape. In contrast, the insertion efficiency of EPL/DAG/1 mol% MPIase LUVs was higher than that expected from its anisotropy value.

From these results, it was concluded that the insertion inhibition by DAG is attributed to the ability to reduce the mobility of the acyl chain in the membrane core region. Although the restored insertion by MPIase is partially attributed to membrane mobility, the increase in insertion efficiency was much larger than expected from the correlation. Additional steps are required for MPIase to achieve higher membrane insertion.

### Interaction of the MPIase pyrophosphate group with arginine and lysine residues in Pf3_27

**Figure 5:**
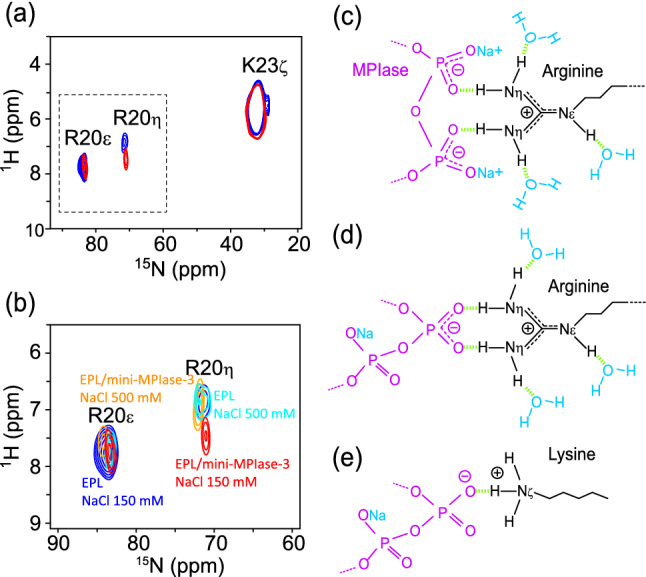
(**a**) Arg and Lys side chain signal regions of the 2D ^1^H-^15^N FSLG-HETCOR spectrum of Pf3_27 (Fig. 1d) in EPL membranes in the absence (blue) and presence (red) of mini-MPIase-3 at 10 °C. The area surrounded by a dashed line is expanded in (**b**). (**b**) Close-up of Arg side chain NH_η_ and NH_ε_ signal regions of 2D ^1^H-^15^N FSLG-HETCOR spectra of ^15^N-labeled Pf3_27 at R20, K23 in the EPL (blue) and EPL/2 mol% mini-MPIase-3 (red) membrane with 150 mM NaCl, and the EPL (light blue) and EPL/2 mol% mini-MPIase-3 (orange) membrane with 500 mM NaCl at 10 °C. (**c**–**e**) Schematic models of Arg (**c** and **d**) or Lys (**e**) side chain (black) interaction with the pyrophosphate linker part of MPIase (purple). Light green dots show hydrogen bonding.

Recently, we demonstrated the importance of a pyrophosphate linker in MPIase for affinity with Pf3 coat protein in an aqueous environment by surface plasmon resonance (SPR) analyses and docking simulations^18^. These results suggest that the pyrophosphate linker of MPIase is one of the key structures in membrane insertion activity. The Pf3 coat protein contains Arg and Lys residues at the C-terminus. Generally, basic amino acid residues in membrane proteins are abundant near the membrane surface in the cytoplasmic domain, known as the positive-inside rule^37,38^. We predicted that the double-negatively charged pyrophosphate group of MPIase would attract the Arg and Lys residues in proteins more strongly than the monophosphate group of other phospholipids. To test this hypothesis, we examined the interaction between the side chains of the basic amino acids and mini-MPIase-3. We used Pf3_27, which has an original C-terminal amino acid sequence around Arg and Lys residues (Fig. 1d), as a substrate in this experiment. Essentially, a proton signal shows a downfield shift when the proton interacts with a negatively charged group^39,40^. Figure 5a and b show the ^1^H-^15^N FSLG-HETCOR spectra of Pf3_27, with ^15^N-labeled at R20 and K23 side chain moieties in EPL multi lamellar vesicles, at 10 °C in the absence (blue) and presence (red) of 2 mol% of mini-MPIase-3. The signal of the R20 H_η_ protons was shifted 0.53 ppm downfield by the addition of mini-MPIase-3, whereas that of the R20 H_ε_ proton did not change. These results indicate that R20 interacted with mini-MPIase-3 at the H_η_ protons (Fig. 5c and d). However, the downfield shift of the K23 H_ζ_ protons was smaller. These results suggest that mini-MPIase-3 interacted more strongly with Arg side chains than with Lys side chains (Fig. 5e). The R20 side chain signal intensities were significantly smaller than those of K23 due to line broadening. This was probably caused by the faster proton exchange of Arg H_η_ and H_ε_ protons than the K23 H_ζ_ protons with water molecules^41^.

Furthermore, we measured the spectra at a NaCl concentration of 500 mM (Fig. 5b, orange and light blue). The signal of the R20 H_η_ protons in the presence of mini-MPIase-3 was almost superimposed with that in the absence of mini-MPIase-3. The intermolecular hydrogen bonds between the pyrophosphate in MPIase and the R20 H_η_ protons of Pf3_27 were cleaved under a high salt concentration. The chemical shift value at NaCl concentrations of 150 and 500 mM was similar without mini-MPIase-3, indicating that intermolecular hydrogen bonds between the monophosphate in membrane lipids and the R20 H_η_ protons of Pf3_27 were less effective.

## Discussion

This study aims to investigate how MPIase and DAG regulate the membrane insertion of a small hydrophobic protein. Solid-state NMR and fluorescence anisotropy techniques were used to analyze the membrane insertion efficiency, tilt angle of the protein, and molecular interaction between MPIase and the protein.

The ^1^H-^15^N SAMPI4 spectra showed that Pf3_24_2 formed an *α*-helical structure in the bicelle ([DMPC]/[DHPC] = 3) with a transmembrane orientation (Fig. 2). This result is in good agreement with the solid-state NMR study of a full-length Pf3 coat protein reconstructed in mechanically aligned POPC lipid bilayers on a glass plate^42^. The tilt angle of Pf3_24_2 determined by SAMPI4 spectra changed only slightly in the presence of mini-MPIase-3 (Fig. 2c). MPIase would interact with proteins only outside and on the surface of the membrane. As the membrane anchor part of MPIase is similar to that of other membrane lipids, MPIase did not have a special impact on the topology of the protein once the protein was embedded inside the membrane.

We evaluated the membrane insertion efficiency of small hydrophobic proteins. Estimation from the amino acid sequence^43^ of Pf3_24 indicated that Pf3_24 exhibited both *α*-helix and *β*-strand propensities and changed the structure depending on the circumstances. The CD spectrum determined an *α*-helical structure in the DMPC LUV (Fig. S12, dark red). However, it was difficult to determine the secondary structure of Pf3_24_3 outside the membrane using CD spectroscopy because Pf3_24_3 precipitated after the formation of aggregates (Fig. S12, blue). In contrast, the solid-state NMR method we proposed here is versatile and could determine the ratio of the amount of the protein inserted into and aggregated outside the membrane when the sample was insoluble.

The strong correlation between the membrane insertion efficiency and the Laurdan GP values or the DPH anisotropy in the bulk membrane suggested that spontaneous membrane insertion depends significantly on the membrane surface packing and the mobility of the acyl chain in the membrane core region (Fig. 4). Both changed drastically as the concentration of the cone-shaped PE increased. It has been reported that the higher lateral pressure in the hydrocarbon core in the cone-shaped lipids in membranes reduces the membrane insertion of the peptides^35,36,44^. Therefore, the high PE content in the EPL membrane may be why the membrane insertion of a substrate protein into the EPL membrane was harder than that into POPC membranes^26^. Furthermore, in this study, the fluorescence experiments indicated that 5 mol% (3.4 w/w%) of DAG, which completely blocked protein insertion in this experiment similar to that in the translational assay, had a significant effect on the mobility of the acyl chain in the membrane core region, but only slightly tightened the membrane surface packing (Fig. 4b), implying that the access of a protein to the membrane core region is more critical than the first contact with the surface region for successful insertion. PE is asymmetrically distributed on the cytoplasmic leaflet of the *E. coli* inner membrane^45^. Therefore, a physiological concentration (~ 1.5 w/w%) of DAG, in combination with PE-rich membrane propensity, can sufficiently block the unregulated spontaneous insertion of proteins into *E. coli*. Meanwhile, eukaryotic cell membranes contain PC, not PE, as the main component. It was reported that 5 mol% of DAG was insufficient to block the insertion into PC membranes^26^. The amount of DAG should be severely limited because DAG serves as a second messenger in signal transduction in eukaryotes^46^. Therefore, cholesterol is involved in blocking spontaneous insertion in eukaryotic cell membranes instead of DAG, although a larger amount (20–30 mol%) is necessary for sufficient blockage^26^.

In contrast, adding an inverted cone-shaped MPIase (Fig. S3) to the DAG-containing membrane would release lateral pressure in the acyl chain region. However, even after adding 1 mol% of MPIase, the reduced mobility by DAG did not completely recover to the intact state of the EPL membrane without DAG (Fig. 4b). Nevertheless, the MPIase-containing EPL/DAG LUVs exhibited efficient insertion.

Inverted cone-shaped Lyso-PC is known to increase membrane insertion of antimicrobial peptides^44^. Addition of Lyso-PC to the EPL/DAG LUVs enhanced the insertion (Fig. S11), but, unlike the enhancement by MPIase, it could be explained only by the alteration of the membrane properties. These results emphasize that the higher integration efficiency of MPIase than expected from the correlation could be attributed to other factors, apart from the membrane properties altered by the inverted cone shape.

Although MPIase significantly recover the membrane insertion in the DAG-containing EPL LUVs, it could not improve insertion in the DAG-free EPL LUV (Fig. 3e,f and Table 2). MPIase did not alter the membrane properties of the DAG-free EPL LUVs, either (Fig. S11). In a previous study, we observed that the interaction rate between mini-MPIase and Pf3 coat protein were accelerated when mini-MPIase-3 was reconstructed in DAG-containing EPL LUVs, suggesting that it assembles on the membrane cooperatively^18^. We also observed that mini-MPIase-3 electrostatically interacts with DAG on the membranes and inhibited the flip-flop motion of DAG^21^. It was reported that gangliosides formed dynamic clusters enriched in cone-shaped molecules like CER and DAG^47^. Therefore, MPIase might also form an assembly in combination with DAG to compensate for their shapes but not in the absence of DAG.

We further emphasize the importance of the pyrophosphate structure in MPIase in this study using the ^1^H-^15^N FSLG-HETCOR spectra, because the pyrophosphate efficiently bound to the Pf3_27 R20 side chain, even in the presence of abundant monophosphate groups of phospholipids on the membrane surface (Fig. 5a,b). Although it could not be concluded whether the Pf3 coat protein binds to one or both the phosphate groups (Fig. 5c,d) in our experiments, Avilés-Moreno et al. reported that a tweezer-like configuration, similar to that shown in Fig. 5c, is more favorable^48^.

A large downfield-shift after complex formation was reported in the analysis of the C-terminus SH2 domain of phospholipase-C_γ_1 (PLCC SH2) with a 12-residue phosphotyrosine-containing peptide (pY1021)^40^. Here, H_η_ protons were non-equivalent and only the centrally situated protons in the guanidino group showed approximately 2–3 ppm downfield shifts. In this study, the downfield shift of the H_η_ protons of Pf3_27 R20 was smaller than that in pY1021. Therefore, we speculated that the binding between the guanidino group of R20 and the pyrophosphate in MPIase is weaker and more transient, compared to that of the ligand-receptor complex.

The downfield shift of K23 H_ζ_ in Pf3_27 was smaller than that of R20 H_η_ (Fig. 5a). Arg residues were more abundant at the interface of a protein–protein or protein-DNA complex compared to Lys residues, due to the higher ability of the guanidinium group in Arg to form a hydrogen bond than the amino group present in Lys^49^. Rohs et al. showed that this difference is attributed to the smaller solvation-free energy of the guanidinium group in Arg residues^50^.

**Figure 6:**
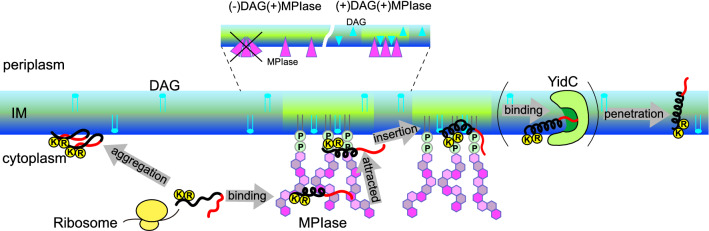
Schematic model of the membrane insertion of a small hydrophobic substrate protein in *E. coli* membranes. Besides the PE-rich propensity of the cytoplasmic leaflet in the inner membrane (dark blue), the presence of DAG (light blue) blocks spontaneous insertion by reducing the mobility of the acyl chains in the membrane core region (dark green). However, in the presence of MPIase together with DAG, the mobility is increased (light green). A protein (black: hydrophobic region, red: hydrophilic region) reaches the membrane surface in an unfolded state following its release from a ribosome. MPIase may assemble with DAG, probably to compensate for their shapes (shown in the close-up of the membrane indicated by dashed lines), and the sugar chain of MPIase captures the protein and inhibits protein aggregation. Subsequently, the side chains of the positively charged residues in the protein are attracted to the membrane surface through the pyrophosphate group of MPIase. This triggers the insertion of the protein into the membrane. The protein easily gains access to the loosened membrane core region. If the protein has a hydrophilic region, it is delivered to the hydrophilic groove of YidC. If the protein is hydrophobic enough, it can be embedded into the membrane without the help of YidC.

Based on the results of this study, we proposed the mechanism for Sec-independent membrane insertion of a small hydrophobic protein containing basic amino acids at its C-terminus, as shown in Fig. 6. Using an antibody, we demonstrated that MPIase is present at the cytoplasmic site of INVs^8^. The trace amount of MPIase and DAG forms a transient assembly probably because of their complementary shapes (shown in the close-up of the membrane indicated by dashed lines in Fig. 6). The flexible long sugar chains interact with the protein and prevent protein aggregation after a substrate protein is released from the ribosomal tunnel^8,10,18^. The double negative charge of the pyrophosphate linker part of MPIase strongly attracts the basic amino acid residues at the membrane surface and immediately delivers the protein into the membrane. Moreover, MPIase increases the mobility of the acyl chain in the membrane core region, leading to the exposure of the hydrophobic acyl chains of membrane lipids to the aqueous environment. This allows the protein to easily gain access to the core region of the membrane. Unless the protein is hydrophobic enough, a hydrophilic region would be delivered to the YidC, enabling the protein to penetrate completely. In the membrane, the protein adopts an *α*-helical conformation. MPIase interacts with the protein via its pyrophosphate group. However, the bond is weak and MPIase can detach from the protein easily. The detached MPIase could assist another substrate that is not yet inserted into the membrane.

In conclusion, this study provides a basic understanding of the role of MPIase in Sec translocon-independent membrane insertion of a small hydrophobic protein into the *E. coli* inner membrane. Combined analyses of the membrane insertion activity, physicochemical properties, and MPIase-substrate intermolecular interaction provide further understanding of the molecular mechanism at the atomic level of how MPIase attracts the substrate near the membrane surface and inserts it.

Further studies on the clustering of the sugar chain in MPIase on the membrane would provide a deeper insight into how MPIase captures a hydrophobic protein synthesized by a ribosome and prevents it from aggregating.

## Material and methods

### Materials

MPIase was purified from MC4100, following the method previously described^9^. An MPIase analog, mini-MPIase-3, was synthesized as previously described^10^. Five types of ^15^N-labeled Pf3 coat protein samples were prepared, including a full-length protein (Pf3_44), transmembrane regions labeled at different residues (Pf3_24_1, Pf3_24_2, and Pf3_24_3), and a transmembrane region followed by a C-terminal sequence (Pf3_27), according to the experiments, as shown in Fig. 1d. Pf3_44, Pf3_24_1, and Pf3_24_2 were synthesized using the fluoren-9-ylmethoxycarbonyl (Fmoc) solid-phase methodology. High performance liquid chromatography (HPLC) and matrix-assisted laser desorption/ionization-time of flight (MALDI-TOF) mass spectrometry (MS) were used to confirm the purity of the proteins, as shown in Figs. S1 and S2, respectively. The preparation of individual proteins is described in the Supplementary Information. Pf3_24_3 and Pf3_27 were custom-made and characterized by Eurofins Genomics K.K. (Tokyo, Japan) and Peptide Institute, Inc. (Osaka, Japan), respectively. *E. coli* polar lipid extract (EPL), 1,2-dimyristoyl-*sn*-glycerol (DMG), 1,2-diheptanoyl-*sn*-glycero-3-phosphocholine (DHPC), 1,2-dimyristoyl-*sn*-glycero-3-phosphocholine (DMPC), 1,2-dimyristoyl-*sn*-glycero-3-phospho-(1'-rac-glycerol) (DMPG), 1-palmitoyl-2-oleoyl-*sn*-glycero-3-phospho-L-serine (POPS), and 1-palmitoyl-2-oleoyl-*sn*-glycero-3-phosphoethanolamine (POPE) were purchased from Avanti Polar Lipids (Alabaster, AL, USA). All lipids were used without further purification. DMG was used as a representative of DAG and referred to as DAG in this study, unless otherwise noted. This was because DAGs possessing C8-C18 fatty acids showed similar blocking activities for membrane protein insertion^7^. Furthermore, 1,6-diphenyl-1,3,5-hexatriene (DPH) and 6-lauroyl-2-dimethylamino naphthalene (Laurdan) were purchased from Sigma-Aldrich (St. Louis, MO, USA). The methodology is discussed in detail in the Supplementary Information.

## Supplementary Information

Supplementary Information.

## Data Availability

The datasets generated during the study are available from the corresponding author upon reasonable request.
