# Supplementary material for: Role of a bacterial glycolipid in Sec-independent membrane protein insertion
Source: Sci Rep. 2022 Jul 18;12:12231. doi: 10.1038/s41598-022-16304-1 (PMC9293918; doi:10.1038/s41598-022-16304-1)
Supplement: Supplementary file 1 — Supplementary Information. [file 41598_2022_16304_MOESM1_ESM.pdf]

Supplementary Information

Role of a bacterial glycolipid in Sec-independent membrane protein insertion

Kaoru Nomura[1,*], Shoko Mori[1,2], Kohki Fujikawa[1], Tsukiho Osawa[1], Shugo Tsuda[3], Kumiko Yoshizawa-Kumagaye[3], Shun Masuda[3], Hideki Nishio[3], Taku Yoshiya[3], Takao Yoda[4], Masafumi Shionyu[4], Tsuyoshi Shirai[4], Ken-ichi Nishiyama[5], Keiko Shimamoto[1,2,*]

[1] Bioorganic Research Institute, Suntory Foundation for Life Sciences, 8-1-1 Seikadai, Seika-cho, Soraku-gun, Kyoto 619-0284, Japan
[2] Department of Chemistry, Graduate School of Science, Osaka University, 1-1 Machikaneyama, Toyonaka, Osaka 560-0043, Japan
[3] Peptide Institute, Inc., 7-2-9 Saito-Asagi, Ibaraki, Osaka 567-0085, Japan
[4] Department of Frontier Bioscience, Nagahama Institute of Bio-Science and Technology, 1266 Tamura-cho, Nagahama, Shiga 526-0829, Japan
[5] Department of Biological Chemistry and Food Sciences, Faculty of Agriculture, Iwate University, 3-18-8 Ueda, Morioka, Iwate 020-8550, Japan

* Corresponding author, E-mail: nomura@sunbor.or.jp; shimamot@sunbor.or.jp

Supplementary Methods

Peptide preparation

Full-length Pf3 (**Pf3_44**) for the ^{15}N 1D cross-polarization nuclear magnetic resonance (CP NMR) experiment (Fig. S4a) was synthesized via automated fluoren-9-ylmethoxycarbonyl (Fmoc) solid-phase peptide synthesis (Fmoc SPPS) on an ABI 433A peptide synthesizer (Applied Biosystems, Bedford, MA, USA) and assembled with the

coupling protocol using Fmoc-amino acid/*N*, *N*-diisopropylcarbodiimide (DIC)/ethyl 2-cyano-2-(hydroxyimino)acetate (OxymaPure®)[1]. Boc-Thr(Fmoc-Val)-OH, an *O*-acyl isodipeptide unit, was introduced into the Val^8-Thr^9 bond[2,3]. After construction of the protected peptide resin, the *O*-acyl isopeptide was cleaved by a trifluoroacetic acid (TFA) cocktail, purified by high-performance layer chromatography (HPLC), and applied to the NMR study with *in situ O*-to-*N* acyl migration (Fig. S1) to produce the native target peptide[4]. The mass spectrum (MS) of the *O*-acyl isopeptide was observed using an Agilent G1956B LC/MSD detector using an Agilent 1100 series HPLC system, and the observed mass (most abundant masses) was derived from the experimental *m/z* value for the protonation state of the target peptide. Electrospray ionization MS: $[M+H]^+$ calculated for $C_{213}H_{351}N_{48}{}^{15}N_5O_{59}S$ was 4634.6, while the measured value was found to be 4634.7.

Pf3_24_1 for the ^{15}N 1D CP NMR experiment (Fig. S4b) and **Pf3_24_2** for the tilt angle analysis carried out in SAMPI4 experiments (Fig. 2) were synthesized by Fmoc SPPS on a CS 336X peptide synthesizer (CSBio Co., Menlo Park, CA, USA). After cleavage from the resin, the peptide was washed several times with diethyl ether and used without further purification. The MS of the peptide was measured using a Bruker Ultraflex III (Bremen, Germany) (Fig. S2). Matrix-assisted laser desorption ionization time-of-flight mass spectrometry (MALDI-TOF-MS): $[M+H]^+$ calculated for $C_{116}H_{199}N_{20}{}^{15}N_9O_{29}$ was 2471.5, while the measured value was found to be 2471.5.

Preparation of the membrane sample

To prepare the membrane sample, 4-(2-hydroxyethyl)-1-piperazineethanesulfonic acid (HEPES) buffer (50 mM HEPES-KOH, 150 mM NaCl; pH 7.5) was used for all experiments except circular dichroism (CD) measurements; phosphate buffer (50 mM

phosphate, 50 mM NaCl; pH 7.4) was used for CD measurements. The bicelle samples for the ^{1}H-^{15}N SAMPI4 (Fig. 2) and ^{15}N 1D CP NMR (Fig. S4) experiments were prepared as follows. Sixty microliters of 1,2-dimyristoyl-*sn*-glycero-3-phosphocholine (DMPC; 34.5 μmol) multi lamellar vesicle (MLV) was mixed with **Pf3_24_1** (Fig. S4b), **Pf3_24_2** (Fig. 2), or **Pf3_44** (Fig. S4a) (0.38 μmol) solubilized with a suspension of 1,2-diheptanoyl-*sn*-glycero-3-phosphocholine (DHPC; 11.5 μmol) in 60 μL of HEPES buffer. After the mixture was vortexed for 15 min at 4 °C, it was placed in a water bath at 37 °C for 30 min. The resulting mixture was extremely viscous but became fluid after shaking it in an ice bath. Repeating the heating (38 °C) and cooling (4 °C) cycles four times resulted in a solution containing 25% w/v bicelles composed of DMPC and DHPC ([DMPC]/[DHPC]=3). Bicelles containing mini-MPIase-3 were synthesized using a similar method from DMPC (33.8 μmol)/mini-MPIase-3 (0.7 μmol, 2 mol% in lipid total amount) MLV.

For the membrane insertion experiments (Figs. 3, 4, S7, S10, and S11), we first prepared large unilamellar vesicles (LUVs). Phospholipids (34.5 μmol) were solubilized in chloroform in the absence and presence of MPIase (mini-MPIase-3 [5 mol% in total lipid amount] or natural MPIase [1 mol% in total lipid amount]) and/or diacylglycerol (DAG; 5 mol% in total lipid amount). After complete solvent evaporation, the resulting lipid film was hydrated with 400 μL of HEPES buffer and vortexed. The suspension was freeze-thawed for ten cycles and extruded through 100-nm polycarbonate filters (Avestin, Ottawa, ON, Canada). Then, **Pf3_24_3** (0.77 μmol) solubilized with 200 μL of DHPC (6.9 μmol) solution was mixed with LUV and incubated for 30 min at 37 °C. This mixture was diluted 20 times with HEPES buffer until the DHPC concentration reached below the critical micelle concentration (CMC) of DHPC (1.6 mM)[5,6]. The supernatant containing

DHPC was removed after centrifugation, and this step was repeated twice to remove DHPC. For all NMR measurements, samples were packed within a 4-mm NMR tube that was closed tightly with a seal cap to prevent drying (Phi Creative, Kyoto, Japan).

To measure fluorescence (Fig. 4, S8, and S9), phospholipids (0.1 μmol for packing analysis or 2.72 μmol for anisotropy measurement) were solubilized in chloroform in the absence and presence of natural MPIase (1 mol% in total lipid amount) and/or DAG (5 mol% in total lipid amount). 6-Lauroyl-2-dimethylamino naphthalene (Laurdan; 1 nmol for packing analysis) or 1,6-diphenyl-1,3,5-hexatriene (DPH; 2 nmol for anisotropy measurement) from an ethanol stock was added before drying the samples. After complete evaporation of the solvent, the lipid film was hydrated with 1 mL of HEPES buffer (final lipid concentration of 0.1 mM) and vortexed extensively. The suspension was freeze-thawed for ten cycles and transformed into 100 nm LUVs using an extruder (Avestin).

For the ^{1}H-^{15}N FSLG-HETCOR experiments (Fig. 5), *Escherichia coli* phospholipid (EPL; 34.5 μmol) or EPL (33.8 μmol)/mini-MPIase-3 (0.7 μmol) solubilized in chloroform and **Pf3_27** (0.7 μmol) solubilized in HFIP were mixed. After complete solvent evaporation, the lipid film was hydrated with 1 mL of HEPES buffer and vortexed extensively. The suspension was freeze-thawed for ten cycles. After centrifugation, the supernatant was removed to obtain a final sample volume of 100 μL.

For the CD measurements (Fig. S12), three types of samples were prepared: (i) **Pf3_24_3** in DMPC LUV. **Pf3_24_3** (0.38 μmol) was co-solubilized with DMPC (17.5 μmol) and LUVs were prepared as described above. (ii) Pf3_24 (0.38 μmol) solution solubilized with DHPC (3.45 μmol). (iii) **Pf3_24_3** (0.38 μmol) in phosphate buffer. The sample was prepared without using LUV as shown in Figure 2a.

Measurement of solid-state NMR

All solid-state NMR measurements were carried out using a Bruker Avance III 600 (Bruker Biospin, AG, Switzerland) equipped with a narrow-bore magnet operated at a ^{1}H resonance frequency of 600 MHz. Data were recorded using a 4-mm E-free triple-resonance magic-angle spinning (MAS) probe. Typical 90° pulse lengths for ^{1}H was 4.0 μs. During acquisition, 62.5 kHz ^{1}H decoupling using the SPINAL-64 scheme[7] was performed for all experiments. ^{1}H-^{15}N CP measurements were performed using a ramped (70%–100%) spin-lock pulse on the ^{1}H channel and a square contact pulse on the ^{15}N channel with a contact time of 800 μs. To determine the topology of **Pf3_24_3**, we used an improved version of SAMMY called SAMPI4[8]. ^{1}H-^{15}N SAMPI4 (Fig. 2) and ^{15}N CP (Fig. S4) spectra were acquired at 30 °C without spinning. We used a ^{1}H B_1 field strength of 47 kHz during the CP and t_1 contact times. The ^{1}H-^{15}N SAMPI4 spectra had a total of 15 t_1 increment and 750 t_2 complex points, with 9600 scans for each t_1 increment. They were assigned using several single-^{15}N-labeled Pf3_24. For the membrane insertion experiments, the 15N CP spectra (Figs. 3, S7, S10, and S11) were acquired at -5 °C under 5-kHz MAS. The signal-to-noise ratio (SNR) of spectral peaks (Figs. 3, S7, S10, and S11) were calculated by the program "sinocal" in Topspin 3.1 (Bruker Biospin, AG, Switzerland). Subsequently, the errors of the membrane insertion efficiency x in Eq. 1 due to the spectral noise were estimated by calculating the difference in x between two cases in which relative peak intensities at 125 ppm (I^{125}) and 120 ppm (I^{120}) fluctuate due to the noise from I^{125}(1+0.5/SNR) and I^{120}(1-0.5/SNR)) to I^{125}(1-0.5/SNR) and I^{120}(1+0.5/SNR). ^{1}H-^{15}N FSLG-HETCOR spectra (Fig. 5) were obtained at 10 °C under 5-kHz MAS by recording 15 t_1 increments and 750 t_2 complex points, with 5000 scans for each t_1 increment. For the ^{1}H-^{15}N FSLG-HETCOR experiment, ^{1}H homonuclear decoupling was

achieved using the FSLG sequence with a transverse field of 71.4 kHz. The ^{1}H chemical-shift scaling factor due to the FSLG sequence was calibrated on the glycine molecule to 0.72. All the temperatures quoted are the calibrated temperatures. The ^{15}N chemical shifts were externally referenced to the methionine amide resonance of *N*-formyl-Met-Leu-Phe-OH (127.9 ppm)[9].

Data analysis of SAMPI4 spectra

SAMPI4 spectra were calculated using scripts available on a website (https://sites.google.com/site/rickcpage/Home/biomolecular-nmr), which were written in MATLAB language (MathWorks, Natick, MA, USA). For all separated local field spectral simulations, the motionally averaged dipolar magnitude ($\nu_{//}$ = 10.735 kHz) and the principal values for the ^{15}N chemical shift tensor (σ_{11} = 57.3 ppm, σ_{22} = 81.2 ppm, σ_{33} = 227.8 ppm) were used[10]. The relative orientation between the chemical shift tensor element σ_{33} and $\nu_{//}$ of the dipolar tensor was taken as 17°. Backbone dihedral angles (ϕ , ψ) of (-65°, -45°) were considered. To incorporate the wobbling motion of the helix axis, we averaged both the chemical shift and dipolar couplings with a Gaussian distribution (width at half-height value of $\pm\ \chi$°).

Steady-state fluorescence measurement

Fluorescence measurements were performed using a Duetta fluorescence spectrophotometer (Horiba Scientific, Kyoto, Japan). The fluorescence emission spectra (Fig. S8) were measured from 380 to 700 nm with an excitation wavelength of 360 nm and a bandwidth of 5 nm at 37 °C. The background measured in HEPES buffer was

subtracted from all emission spectra. The generalized polarization (GP) of Laurdan was calculated using the following equation:

$$GP = (I_{440} - I_{490})/(I_{440} + I_{490}) \quad \text{(S1)}$$

where I_{440} and I_{490} are the intensities at 440 nm and 490 nm of the emission spectrum, respectively.

In the DPH anisotropy measurements (Fig. S9), the excitation and emission wavelengths were 359 and 426 nm, respectively. Anisotropy was recorded at 5 °C intervals in the range of 2–57 °C. An equilibration time of 5 min was allowed after each temperature change. The steady-state fluorescence anisotropy (r_s) values were calculated as follows:

$$r_s = \frac{I_{VV} - GI_{VH}}{I_{VV} + 2GI_{VH}} \quad \text{(S2)}$$

where I_{VV} and I_{VH} are the emission intensities measured in the parallel and perpendicular directions to the exciting beam, respectively, and G is the grating factor ($G = I_{VH}/I_{HH}$). The addition of 0.1 mol% DPH to EPL LUV increased membrane insertion by 3%. Thus, actual fluorescence anisotropy values on the horizontal axis in the correlation plot (Fig. 4b) are expected to shift almost uniformly and infinitesimally to the smaller value in the absence of DPH, but the slope of the global linear fits would hardly be affected.

CD spectroscopy

CD spectra of **Pf3_24_3** (Fig. S12) were recorded at 37 °C on a Jasco J-725 spectropolarimeter (Jasco, Tokyo, Japan) using a 1-mm-path-length cell. The spectra were measured between 190 and 250 nm, and the average blank spectra were subtracted. Data were collected at 0.1 nm with a scan rate of 100 nm/min and a time constant of 0.5 s. The peptide concentration was 383 μM. Eight scans were averaged for each sample, and the

appropriate background contribution was determined.

Molecular dynamics simulation

We explored MPIase conformation (Fig. S3) using all-atom molecular dynamics (MD) simulations. The system contained four MPIase molecules, a membrane composed of 156 1-palmitoyl-2-oleoyl-*sn*-glycero-3-phosphocholine (POPC) molecules, water (13,717 molecules), and ions (52 K^+ and 36 Cl^-) dissolved in water. The MPIase molecule in the system was composed of two trisaccharide units, {Fuc4NAc(α1-4)ManNAcA(β1-4)GlcNAcA(α1-4)}^{2}, linked to DAG through pyrophosphate. The topology information and initial configuration were constructed using the CHARMM software (ver. 42b1)[11], CHARMM scripts provided by CHARMM-GUI[12], and some manual modifications. Four MPIase molecules were embedded into one of the two leaflets of the membrane. We conducted potential-energy minimization (EM) and MD simulations using the GENESIS software package (ver. 1.1.0)[13] with the CHARMM C36 force field[14] and the TIP3P water model[15]. After a 10,000-steps EM, we conducted a 50-ps NVT MD (MD-1), 25-ps NPT MD (MD-2), 200-ps NPT MD (MD-3), and 100-ps NPT MD (MD-4) to equilibrate the system. Then a 110-ns NPT MD (MD-5) was conducted. In EM and MDs-1 to -3, the torsion angles were restrained to maintain the shape of the sugar rings and the *cis* geometry of the double bonds in the acyl chains of lipids. We also restrained the improper torsion angles centered at the second carbon of glycerol in the lipids. To maintain the shape of the membrane, we restrained phosphorus atoms in the phosphate group bound to glycerol in lipids in the direction of the membrane normal. The MD time step was set to 1 fs in MDs-1, -2, and 2 fs in MD-3 and later. Bonds between heavy and hydrogen atoms were constrained by SHAKE[16] (in MDs -1 to -4) or RATTLE[17] (in MD-5). The target temperature and pressure were set to 310 K and 1.0 atm, respectively. Electrostatic

interactions were calculated using the PME algorithm[18]. The coordinates were stored every 1-ns in the final 100-ns of the MD-5 trajectory.

Supplementary Figures and Tables

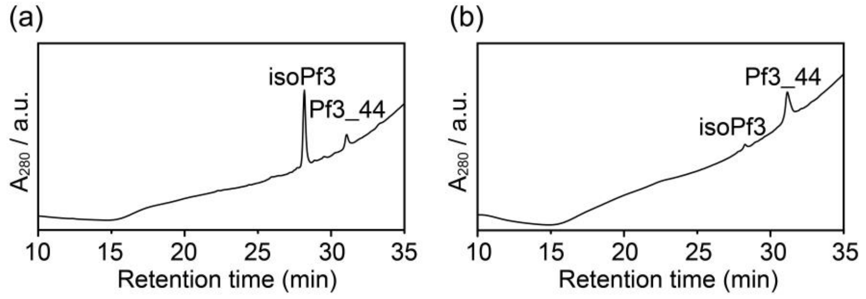

Figure S1. HPLC profile of 9-*O*-acyl isoPf3 (a) and full-length Pf3 (**Pf3_44)** after *in situ* *O*-to-*N* acyl migration (b), analyzed using a Shimadzu Prominence-I LC-2030C (Kyoto, Japan) on a COSMOCIL 5C4-MS column (4.6 × 150 mm) at 60 °C. Solvent A: 0.1% TFA, Solvent B: IPA/CH_3CN/H_2O (6/3/1) in 0.1% TFA, gradient: 40% solvent B over 5 min, linear gradient 40–98% Solvent B over 25 min, and 98% solvent B over 5 min. t_R = 28.2 min in (a) and t_R = 31.1 min in (b). The detection wavelength was 220 nm. The purity of **Pf3_44** was 93%.

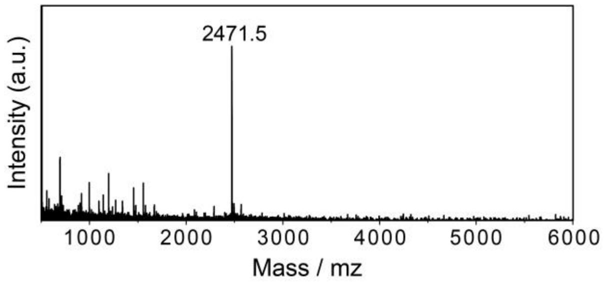

Figure S2. MALDI-TOF-MS spectra of synthesized **Pf3_24_2**.

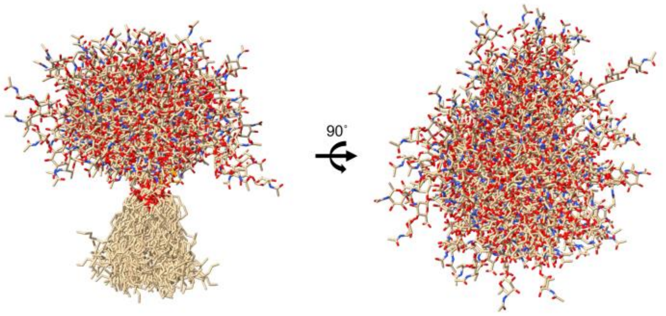

Figure S3. An example of superimposition of 100 conformations of an MPIase molecule. The MD system contained four MPIase molecules, which were composed of two trisaccharide units, pyrophosphate, and DAG. The conformations for each MPIase molecule were extracted from the final 100 ns of the MD trajectory after every 1 ns. All conformations extracted from the MD trajectory were superimposed on the first conformation using the atomic coordinates of pyrophosphate and DAG. Similar inverted cone-shaped structures shown in this figure were also observed for the other three MPIase molecules in the MD system.

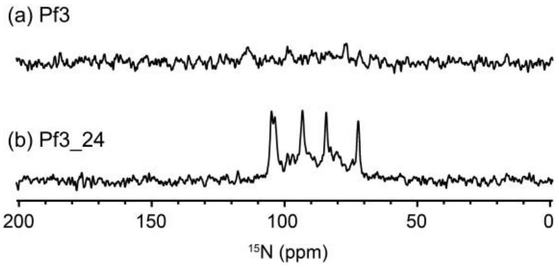

Figure S4. ^{15}N 1D CP spectra of full-length Pf3 (**Pf3_44)** (a) and **Pf3_24_1** (b) added to DMPC/DHPC bicelles ([DMPC]/[DHPC] = 3). Signals of **Pf3_44** were very weak, implying that only a small amount of **Pf3_44** were inserted into the membrane. In contrast, **Pf3_24_1** showed sharp signals originating from ^{15}N-labeled residues, suggesting that

Pf3_24_1 was efficiently inserted into the bicelles at a certain angle. Both spectra were measured without sample spinning at 30 °C.

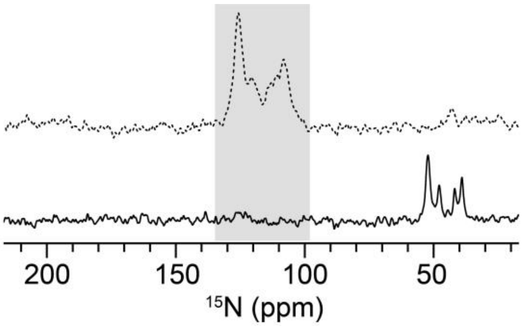

Figure S5. ^{15}N CPMAS NMR spectrum (bottom) of the lyophilized supernatant removed after centrifugation in step (iii) in the procedure shown in Fig. 3a. The **Pf3_24_3** spectrum of the aggregates was represented by the dashed line (top). The absence of signals that originated from **Pf3_24_3** (gray-colored region) in the bottom spectrum indicated that proteins were completely precipitated by centrifugation. The signals at 50 ppm in the bottom spectrum are attributed to HEPES in the dilution buffer.

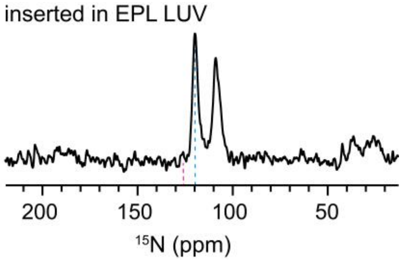

Figure S6. ^{15}N CPMAS NMR spectrum of **Pf3_24_3** reconstituted into the EPL LUV. The membrane insertion procedure shown in Fig. 3a was omitted for this sample. The pink and light blue dashed lines show the chemical shift at 125.6 and 119.6 ppm, respectively.

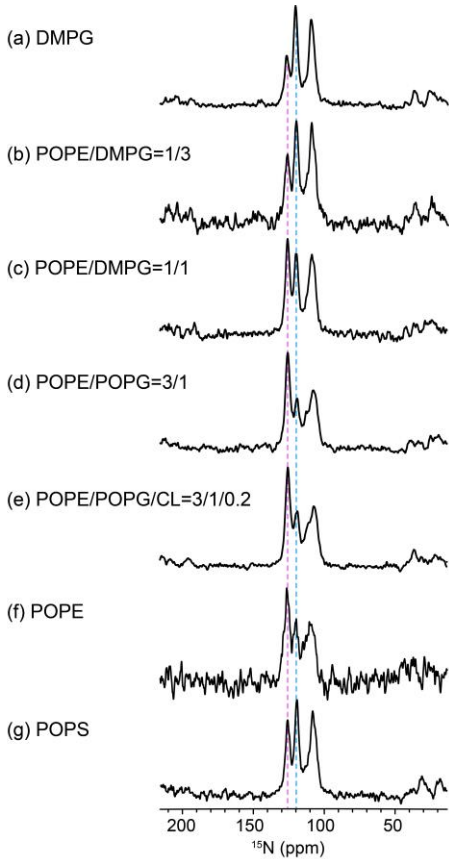

Figure S7. ^{15}N CPMAS NMR spectra of **Pf3_24_3** obtained using the procedure shown in Fig. 3a. LUVs were composed of DMPG (a), POPE/DMPG = 1/3 (b), POPE/DMPG = 1/1 (c), POPE/DMPG = 3/1 (d), POPE/DMPG/CL = 3/1/0.2 (e), POPE (f), and POPS (g). We chose phospholipids with T_m values that were below the assay temperature (37 °C) and comparable to that of DMPC because the acyl chains of phospholipids are ordered below the phase transition temperature T_m. The pink and light blue dashed lines show the chemical shift at 125.6 and 119.6 ppm, respectively.

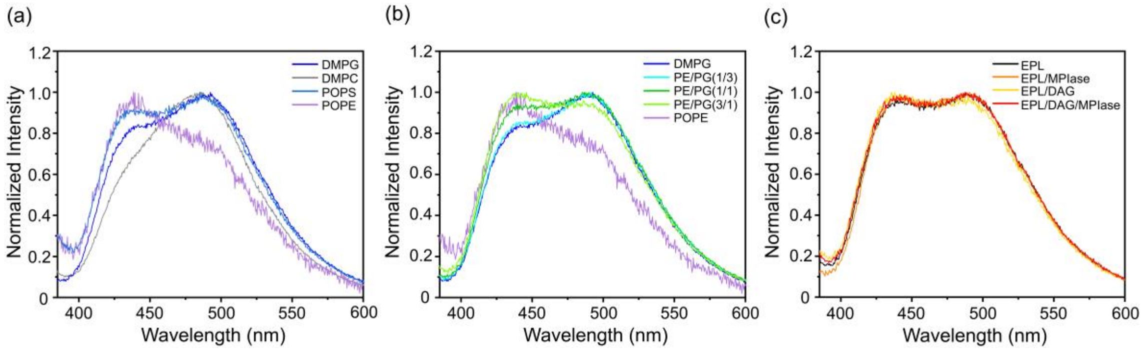

Figure S8. Fluorescence emission spectra of Laurdan incorporated into LUVs composed of (a) DMPG (dark blue), DMPC (gray), POPS (blue), and POPE (purple), (b) DMPG (dark blue), POPE/DMPG (1/3) (light blue), POPE/DMPG (1/1) (green), POPE/DMPG (3/1) (light green), and POPE (purple), (c) EPL (black), EPL/MPIase (99/1) (orange), EPL/DAG (95/5) (yellow), and EPL/DAG/MPIase (94/5/1) (red). All spectra were measured at an excitation wavelength of 360 nm at 37 °C. Data are presented as the means of five independent experiments and normalized to the strongest peak intensity.

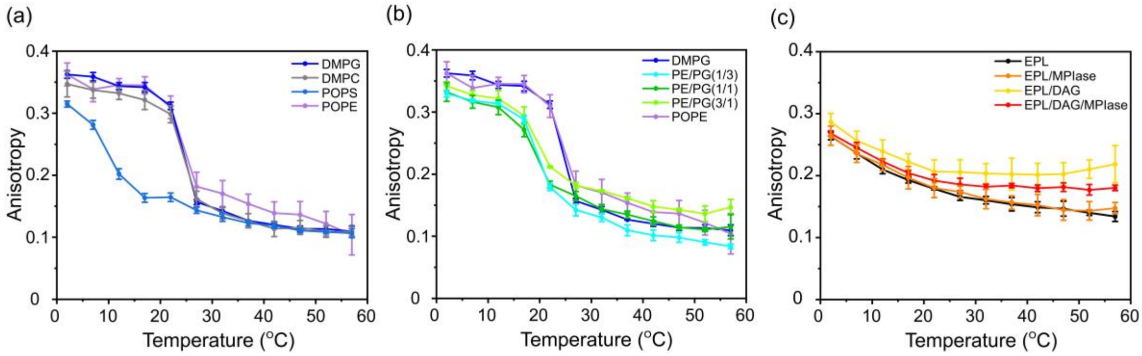

Figure S9. Fluorescence anisotropy of DPH incorporated into various types of LUVs as a function of temperature. Lipid compositions of LUVs were the same as those used in the fluorescence emission experiments shown in Fig. S8. The measured samples were excited by polarized light at 350 nm and emission was monitored at 420 nm. The error bars show the standard deviation of more than three experiments.

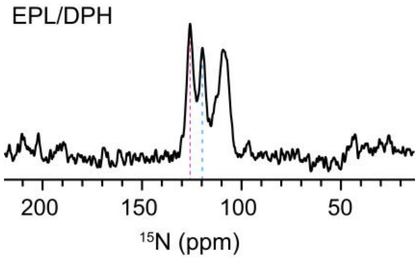

Figure S10. ^{15}N CPMAS NMR spectra of **Pf3_24_3** obtained using the procedure shown in Fig. 3a. LUVs were composed of EPL/DPH (100/0.1). The pink and light blue dashed lines show the chemical shift at 125.6 and 119.6 ppm, respectively.

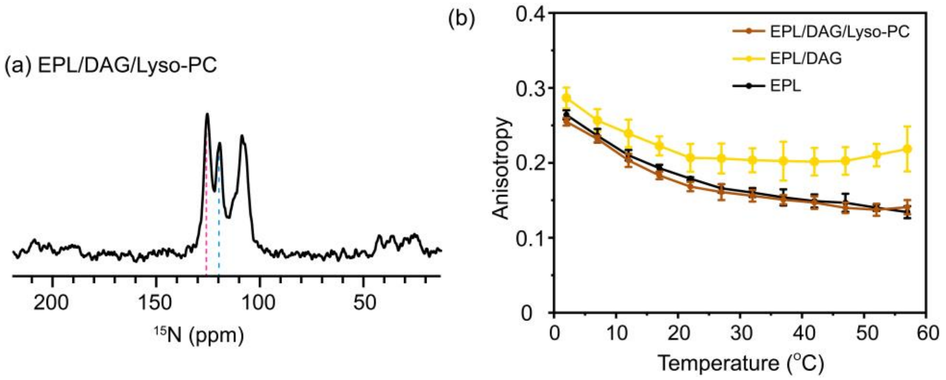

Figure S11. (a) ^{15}N CPMAS NMR spectra of **Pf3_24_3** obtained using the procedure shown in Fig. 3a. LUVs were composed of EPL/DAG/Lyso-PC (90/5/5). The pink and light blue dashed lines show the chemical shift at 125.6 and 119.6 ppm, respectively. (b) Fluorescence anisotropy of DPH incorporated into EPL/DAG/Lyso-PC (90/5/5) (brown), EPL/DAG (95/5) (yellow), and EPL (black) LUVs as a function of temperature. The error bars show the standard deviation of more than three experiments.

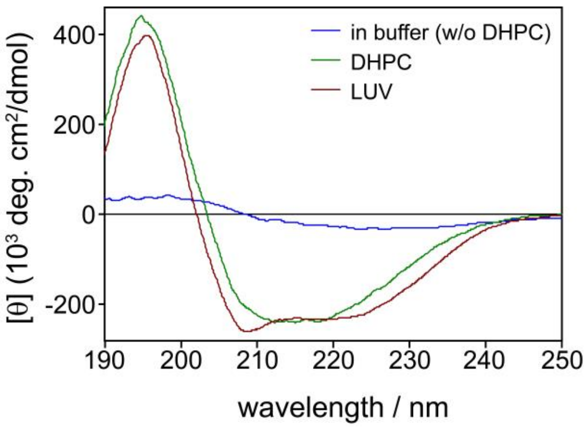

Figure S12. Superimposition of CD spectra of **Pf3_24_3**: **Pf3_24_3** embedded in DMPC LUV, which was co-solubilized with DMPC before making the LUV membranes (dark red). Solubilized with a DHPC solution (green); **Pf3_24_3** in HEPES buffer (blue), which was prepared without using LUVs, as shown in Fig. 2a. These spectra were obtained after subtraction of the background CD spectra. All measurements were recorded at 37 °C.

Table S1. Relative signal intensities at 125 ppm (I^{125}) and 120 ppm (I^{120}) shown in Fig. S7, and the insertion efficiency of Pf3_24, x, into the various membranes.

	I^{125}	I^{120}	Insertion efficiency x (%)†
DMPG	32	68	59 ± 4
POPE/DMPG (1/3)	39	61	50 ± 3
POPE/DMPG (1/1)	54	46	31 ± 1
POPE/DMPG (3/1)	71	29	9 ± 1
POPE/DMPG/CL (3/1/0.2)	67	33	13 ± 1
POPE	65	35	16 ± 5
POPS	44	56	43 ± 2

† x values were calculated using Eq. 1. Errors were estimated from the noise in each spectrum in Fig. S7 using the method described in Supplementary Methods.

Table S2. Relative signal intensities at 125 ppm (I^{125}) and 120 ppm (I^{120}) shown in Figs. S10 and S11 and the insertion efficiency of Pf3_24, x, into the various membranes.

	I^{125}	I^{120}	Insertion efficiency x (%)†
EPL/DPH (100/0.1)	32	68	27 ± 4
EPL/DAG/Lyso-PC (90/5/5)	54	46	26 ± 1

† x values were calculated using Eq. 1. Errors were estimated from the noise in each spectrum in Figs. S10 and S11 using the method described in Supplementary Methods.

Supplementary References

1 Subirós-Funosas, R., Prohens, R., Barbas, R., El-Faham, A. & Albericio, F. Oxyma: an efficient additive for peptide synthesis to replace the benzotriazole-based HOBt and HOAt with a lower risk of explosion. *Chemistry* **15**, 9394-9403 (2009).

2 Sohma, Y. *et al.* 'O-Acyl isopeptide method' for the efficient synthesis of difficult sequence-containing peptides: use of 'O-acyl isodipeptide unit'. *Tetrahedron Lett.* **47**, 3013-3017 (2006).

3 Yoshiya, T. *et al.* "O-acyl isopeptide method" for peptide synthesis: synthesis of forty kinds of "O-acyl isodipeptide unit" Boc-Ser/Thr(Fmoc-Xaa)-OH. *Org. Biomol. Chem.* **5**, 1720-1730 (2007).

4 Sohma, Y. *et al.* Development of O-acyl isopeptide method. *Biopolymers* **88**, 253-262 (2007).

5 Hauser, H., Guyer, W., Pascher, I., Skrabal, P. & Sundell, S. Polar group conformation of phosphatidylcholine. Effect of solvent and aggregation. *Biochemistry* **19**, 366-373(1980).

6 Tausk, R. J., Karmiggelt, J., Oudshoorn, C. & Overbeek, J. T. Physical chemical studies of short-chain lecithin homologues. I. Influence of the chain length of the fatty acid ester and of electrolytes on the critical micelle concentration. *Biophys. Chem.* **1**, 175-183 (1974).

7 Fung, B. M., Khitrin, A. F. & Ermolaev, K. An improved broadband decoupling sequence for liquid crystals and solids. *J. Magn. Reson.* **142**, 97-101 (2000).

8 Nevzorov, A. A. & Opella, S. J. Selective averaging for high-resolution solid-state NMR spectroscopy of aligned samples. *J. Magn. Reson.* **185**, 59-70 (2007).

9 Rienstra, C. M. *et al.* De novo determination of peptide structure with solid-state magic-angle spinning NMR spectroscopy. *Proc. Natl. Acad. Sci.* **99**, 10260-10265 (2002).

10 Wang, J. *et al.* Imaging membrane protein helical wheels. *J. Magn. Reson.* **144**, 162-167 (2000).

11 Brooks, B. R. *et al.* CHARMM: the biomolecular simulation program. *J. Comput. Chem.* **30**, 1545-1614 (2009).

12 Jo, S., Kim, T., Iyer, V. G. & Im, W. CHARMM-GUI: a web-based graphical user interface for CHARMM. *J. Comput. Chem.* **29**, 1859-1865 (2008).

13 Kobayashi, C. *et al.* GENESIS 1.1: A hybrid-parallel molecular dynamics simulator with enhanced sampling algorithms on multiple computational platforms. *J. Comput. Chem.* **38**, 2193-2206 (2017).

14 Klauda, J. B. *et al.* Update of the CHARMM all-atom additive force field for lipids: validation on six lipid types. *J. Phys. Chem. B* **114**, 7830-7843 (2010).

15 Jorgensen, W. L., Chandrasekhar, J. & Madura, J. D. Comparison of simple potential functions for simulating liquid water. *J. Chem. Phys.* **79**, 926-935 (1983).

16 Ryckaert, J. P., Ciccotti, G. & Berendsen, H. J. C. Numerical integration of the cartesian equations of motion of a system with constraints: molecular dynamics of n-alkanes. *J. Comput. Phys.* **23**, 327-341 (1977).

17 Andersen, H. C. Rattle: A"velocity" version of the Shake Algorithm for molecular dynamics calculations. *J. Comput. Phys.* **52**, 24-34 (1983).

18 Essmann, U., Perera, L. & Berkowitz, M. L. A smooth particle mesh Ewald method. *J. Chem. Phys.* **103**, 8577-8593 (1995).
